# Supplementary material for: ALDH2 p.E504K Variation and Sex Are Major Factors Associated with Current and Quitting Alcohol Drinking in Japanese Oldest Old
Source: Genes (Basel). 2021 May 24;12(6):799. doi: 10.3390/genes12060799 (PMC8225027; doi:10.3390/genes12060799)
Supplement: Supplementary file 1 [file genes-12-00799-s001.zip › genes-1185399-supplementary.pdf]

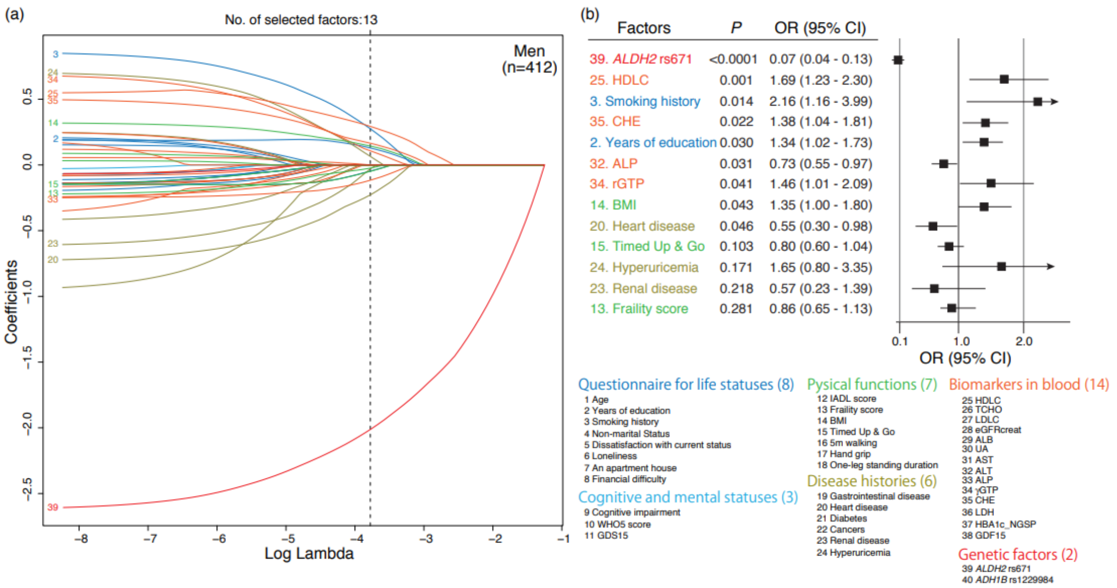


**Supplementary Figure 1.** Variable selection associated with current drinking by LASSO and its multivariate regression logistic analysis against Oldest Old men.(a) Variable selection associated with current drinking by LASSO in Oldest Old men. Five-fold cross-validation to determine an optimal parameter lambda selected 13 factors from 41 factors for further analysis. (b) Multivariate regression logistic analysis for current drinking and LASSO selected 13 factors in Oldest Old men.

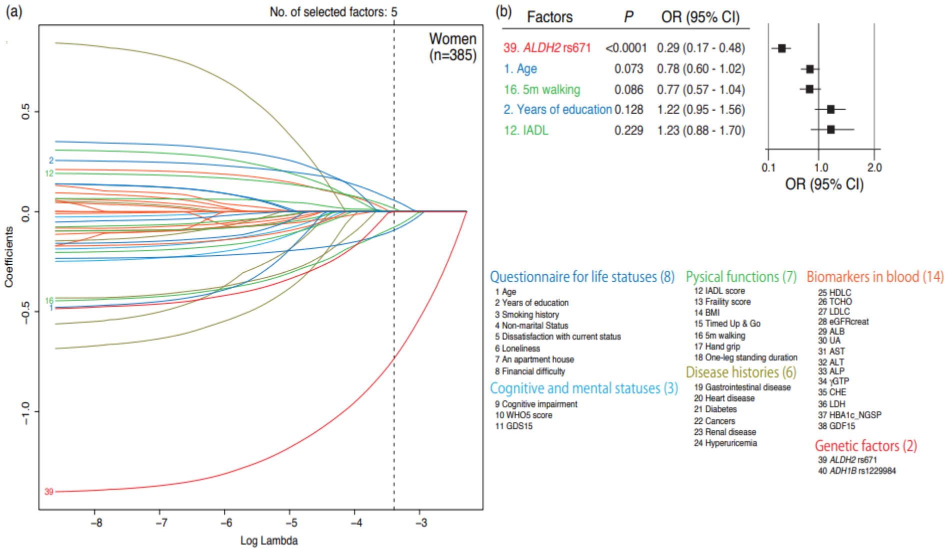


**Supplementary Figure 2.** Variable selection associated with current drinking by LASSO and its multivariate regression logistic analysis against Oldest Old women(a) Variable selection associated with current drinking by LASSO in Oldest Old women. Five-fold cross-validation to determine an optimal parameter lambda selected 5 factors from 41 factors for further analysis. (b) Multivariate regression logistic analysis for current drinking and LASSO selected 5 factors in Oldest Old women.
